# Risk Factors for Introduction of Bovine Herpesvirus 1 (BoHV-1) Into Cattle Herds: A Systematic European Literature Review

**DOI:** 10.3389/fvets.2021.688935

**Published:** 2021-10-27

**Authors:** H. W. Frederik Waldeck, Linda van Duijn, Kristel van den Heuvel-van den Broek, Maria H. Mars, Inge M. G. A. Santman-Berends, Marit M. Biesheuvel, Gerdien van Schaik

**Affiliations:** ^1^Royal GD, Deventer, Netherlands; ^2^Department of Production Animal Health, Faculty of Veterinary Medicine, University of Calgary, Calgary, AB, Canada; ^3^Department of Population Health Sciences, Faculty of Veterinary Medicine, Utrecht University, Utrecht, Netherlands

**Keywords:** BoHV-1, IBR/IPV/IPB, introduction, risk factor, epidemiology, eradication, biosecurity, review

## Abstract

Given that bovine herpesvirus 1 (BoHV-1) -the causative agent of Infectious Bovine Rhinotracheitis (IBR)- is still endemic in most European countries, BoHV-1 free herds are subject to a considerable risk of (re)introduction of the virus. The aim of this literature review was to describe published, quantified risk factors that are relevant for the introduction of BoHV-1. The risk factors described in this study can be used as input for modeling eradication scenarios and for communication on biosecurity measures to stakeholders. A literature search was conducted in November 2020 in two major online search databases, PubMed and Web of Science. The search criteria “risk factor” combined with different synonyms for BoHV-1 were explored, which resulted in 564 hits. Only studies performed in Europe, written in Dutch, English, French, German or Spanish with an English summary and that quantified risk factors for introduction of BoHV-1 into cattle herds were included. Studies had to quantify the risk factors with crude odds ratios (OR), an estimate of the chance of a particular event occurring in an exposed group to a non-exposed group. After checking for duplicates and excluding articles that did not meet the inclusion criteria, 12 publications remained for this review. Risk factors were classified into seven groups, i.e., herd characteristics, management, animal characteristics, purchase, direct animal contact, neighborhood and indirect transmission routes. Most relevant factors for introduction of BoHV-1 into cattle herds include herd size, purchase of cattle, cattle density, age of cattle, distance to neighboring cattle herds and professional visitors. Together with other direct and indirect animal contacts, these factors are important when elimination of BoHV-1 is considered. A closed farming system and protective clothing for professional visitors can eliminate the major routes of introduction of BoHV-1 in cattle herds. To the best of our knowledge, this is the first systematic review solely focussing on measures that can be taken to control introduction of BoHV-1 into cattle herds. Besides testing, focus on managing these (biosecurity) factors will decrease the risk of introducing the virus.

## Introduction

Bovine herpesvirus 1 (BoHV-1), the causative agent of Infectious Bovine Rhinotracheitis (IBR), Infectious Pustular Vulvovaginitis (IPV) and Infectious Pustular Balanoposthitis (IPB), is an important viral pathogen of cattle and is found worldwide. It is listed as notifiable by the World Organization for Animal Health (OIE). BoHV-1 generates losses in (sub)clinically diseased cattle and may result in trading restrictions both within and between countries. Although the first reports date back to the 19th century in Germany, the virus detected in the 1950s in feedlots in the western United States of America was named BoHV-1. Through cattle trade (including semen and embryos) the virus was introduced in Europe in 1960. Within a decade, the virus had become endemic in most countries. However, BoHV-1 is over the years successfully eradicated in several European countries or regions, i.e., Austria (1999), Czech Republic (2020), Denmark (1991), Finland (1994), Germany (2017), two provinces/autonomous regions in Italy (Bolzano 2000/Valle d'Aosta 2015), Channel Island Jersey of United Kingdom (2012), Norway (1994), Sweden (1998) and Switzerland (1993). Member states of the European Union (EU) are considered BoHV-1 free officially under EU legislation (directive 1964/432/EEC). Other countries implemented an EU-approved programme, obligatory for cattle herds at a national (i.e., Belgium, France and Luxembourg) or regional level (Italy). Also, some EU member states have BoHV-1 control programmes that are not officially EU-approved but aim to control the virus (e.g., Ireland, the Netherlands, Spain).

On April 21st, 2021, new EU regulation and its delegated acts (directive 2016/429) on transmissible animal diseases went into force, also known as the Animal Health Law (AHL). This new legal framework lays down the rules for disease surveillance, eradication programmes, and disease freedom of several listed diseases, including IBR and potentially, will lead to more focus on the epidemiology of BoHV-1 in other EU member states considering eradication. For BoHV-1 different diagnostic protocols are accepted, in different matrices, i.e., blood and milk, but all focus on eliminating latently infected cattle. To grant a country or region official disease freedom, vaccination has to be banned for at least 2 years, and with 95% confidence 99.8% of herds and 99.9% of cattle ought to be BoHV-1 free.

Knowing which risk factors are objectively relevant and irrelevant for (re)introduction of the virus in cattle herds is essential information for designing effective control programmes (CP) and for communication about BoHV-1 elimination to stakeholders. Quantitative data on probabilities of introduction of BoHV-1 is needed as input for decision support models that evaluate the epidemiological potential of different CP scenarios as basis for national eradication CPs. Furthermore, translating these risk factors into biosecurity measures (defined as all measures that prevent or reduce the introduction of an agent or, if once introduced, can minimize the spread within a herd) for farmers, veterinarians and other professional visitors in the cattle industry is crucial. Addressing risk factors in an applied and evidence-based manner and emphasizing the need and purpose of biosecurity measures to minimize the risk of contracting BoHV-1 infection can help understanding and adoption of these measures to stop the virus from spreading.

Introduction and spread of BoHV-1 mainly occurs through direct animal contacts between susceptible and infected cattle. Many different studies have identified risk factors for BoHV-1 infection, but to our knowledge, the findings of these papers have never been summarized. By bundling the dispersed information of different studies, this systematic literature review provides an overview of the most important risk factors for introducing BoHV-1 in cattle herds in Europe. Solely studies from European countries were evaluated to make results most applicable to the cattle situation in Europe.

## Materials and Methods

A literature search was carried out in the search databases PubMed and Web of Science in November 2020. As search criteria “risk factor” in combination with different synonyms for BoHV-1 were used:

(BHV *or* BHV-1 *or* BoHV *or* BoHV-1 *or* Bovine herpesvirus *or* IBR *or* IBRV *or* Infectious Bovine Rhinotracheitis).

The retrieved reference management files were exported to Covidence (Veritas Health Innovation, Melbourne, Australia). This web-based software platform enabled two authors (HW and LvD) to independently systematically review by title, abstract and full text screening to determine eligibility against the review inclusion criteria.

**Figure 1:**
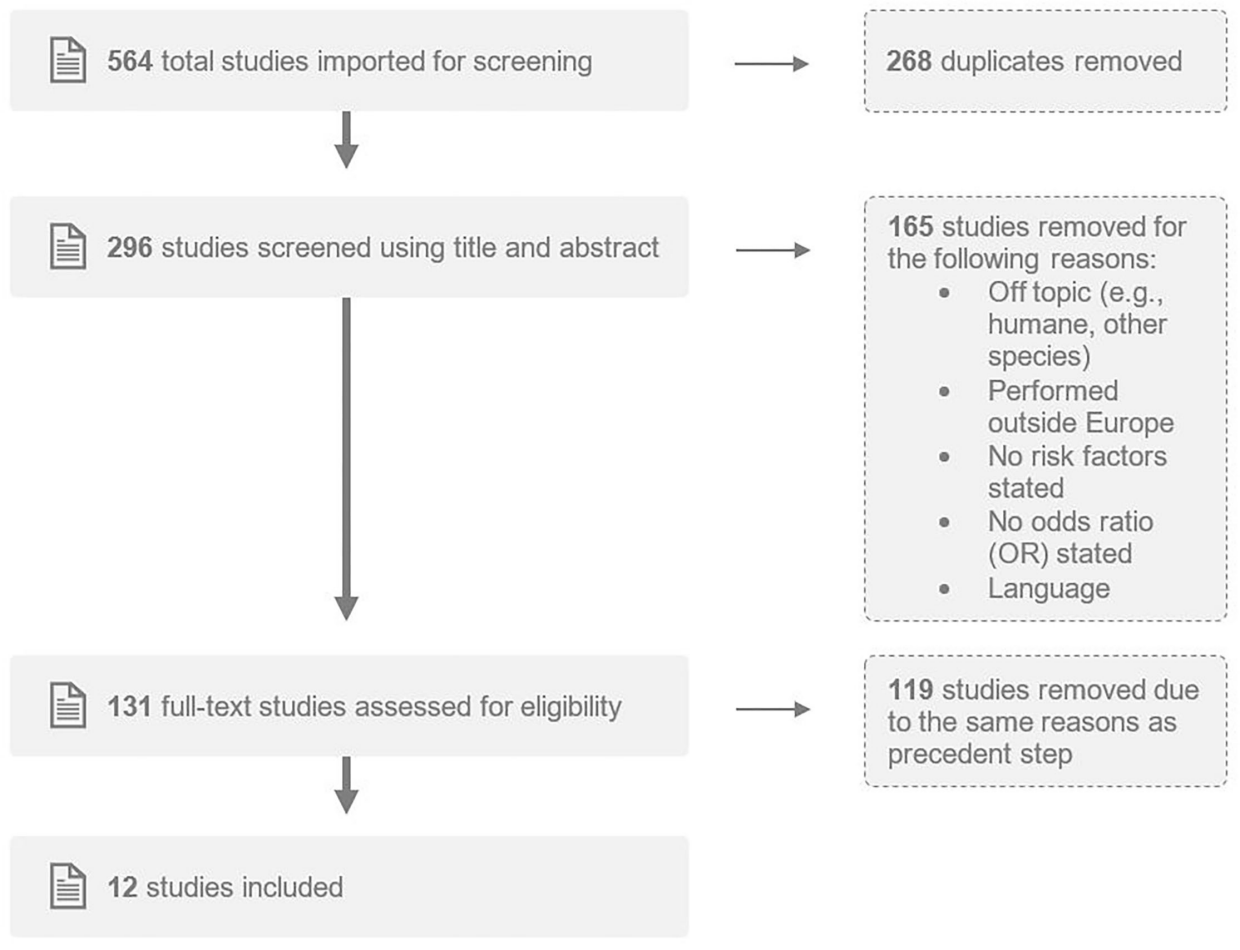
Flow chart of studies through the selection process within the systematic review.

The search amounted to 564 hits and after removal of duplicates 296 publications remained. Only studies performed in Europe, written in Dutch, English, French, German or Spanish with an English summary and that quantified risk factors for introduction of BoHV-1 into cattle herds were included. After removing articles irrelevant to the topic and excluding articles that did not meet the inclusion criteria by title and abstract screening, the remaining 131 studies' full-texts were assessed for further inclusion. Subsequently, the categorization of the two authors was compared and discussed for definite approval. Finally, the first author reported on 12 studies and relevant results are included in this paper (see Figure 1 for details).

Findings were listed when a reviewed study used crude odds ratios (OR) to quantify the risk factor for introduction of BoHV-1. An OR is an estimate of the chance of a particular event occurring in an exposed group to its rate of occurrence in a non-exposed group. For all risk factors, significance was assumed when the *p*-value (p) was 0.05 or below, and both the point estimate and the corresponding 95% confidence intervals (CI) are presented when available.

## Results

The review includes 12 studies from six different European countries: Belgium (BE: 1), Estonia (EE: 1), Ireland (IE: 3), the Netherlands (NL: 4), Spain (ES: 1) and the United Kingdom (UK: 2). Studies could have different study designs, but they all quantified risk factors by OR. The magnitude of the effect differed between studies. Some characteristics of the studies are presented in Table 1.

**Table 1:** Characteristics of the 12 reviewed studies.

**References**	**Year**	**Co**.	**Study period**	**#Herds**	**Herd type**	Matrix^*^	**Serostatus**
Bishop et al. (1)	2010	UK	2/2008-5/2008	50	Dairy	BM	Herd
Boelaert et al. (2)	2005	BE	1998	309	Beef/dairy	11.284 BS	Animal/herd
Gonzalez-Garcia et al. (3)	2009	ES	1/2000-4/2000	110	Beef/dairy	2.393 BS	Animal/herd
Martinez-Ibeas et al. (4)	2015	IE	2009	305	Dairy	BM + 529 BS	Animal/herd
O'Grady at al. (5)	2008	IE	11/2007	41	Beef	BS	Herd
Raaperi et al. (6)	2010	EE	9/2006-4/2008	103	Dairy	BM + 9.637 BS	Animal/herd
Sayers et al. (7)	2015	IE	2009	305	Dairy	BM + 2.171 BS	Animal/herd
Van Schaik et al. (9)	1998	NL	2/1996-4/1996	107	Dairy	BM+BS	Animal/herd
Van Schaik et al. (10)	2001	NL	3/1997-4/1999	119	Dairy	BM+BS	Animal/herd
Van Schaik et al. (11)	2002	NL	3/1997-4/1999	95	Dairy	BM	Herd
Van Wuijckhuise et al. (12)	1998	NL	11/1994	32.955	Dairy	BM	Herd
Williams and Van Winden (13)	2014	UK	12/2008-3/2010	1.088	Dairy	BM	Herd

* *BM, bulk milk; BS, blood samples (the number indicates the amount of blood samples when available)*.

The findings on different risk factors were classified into seven groups, i.e., herd characteristics, management, animal characteristics, purchase, direct animal contact, neighborhood and indirect transmission routes. The most important results on OR are summarized in Table 2.

**Table 2:** Summary of studied risk factors for introducing BoHV-1 into cattle herds.

**Risk factor (RF)**	**#Studies reported RF**	**#Studies effect RF**	**Range OR (*p* < 0.05)**
**HERD FACTORS**			
Herd size	10	8	1.005–14.57
Herd type	3	2	1.9–4.84
**MANAGEMENT FACTORS**			
Seasonal calving	1	0	–
Presence of a bull	3	2	1.52–2.13
Borrowing machinery	1	0	–
**AMINAL FACTORS**			
Breed	2	1	7.91
Sex (M>F)	2	2	1.14–1.37
Age	4	3	1.04–28.94
**PURCHASE RELATED FACTORS**			
Purchase of cattle	10	7	1.32–16.7
Rejected export cattle	2	1	12.6
**ANIMAL CONTACT FACTORS**			
Cattle shows	4	1	3.54
(Communal) grazing	5	2	3.07–7.0
Housing	1	0	–
Other species	1	0	–
**NEIGHBORHOOD FACTORS**			
Herd density	6	3	1.13–2.8
Distance between herds	3	2	1.43–7.58
Escaping and mingling	3	1	6.85
**INDIRECT RISK FACTORS**			
Visitors	5	3	4.06–6.05
Vaccination	2	0	–

*Column I of Table 2 indicates the risk factor, column II states the number of studies that reported on the risk factor (out of 12), column III states the number of studies that mention a significant association of the risk factor, and column IV provides the range in odds ratios (OR) from the studies that reported an effect*.

### Herd Factors

#### Herd Size

The association between herd size and the presence of BoHV-1 in cattle herds was evaluated in ten studies. In eight of those, larger herds were found BoHV-1 positive significantly more often than smaller herds.

Raaperi et al. (6) found that the herd prevalence (antibodies to BoHV-1) of Estonian dairy herds increased with herd size, being 3.4% in the smallest herds (<20 cows) and 85.7% in large herds (over 400 cows). A significant increase in prevalence was seen when herd size categories 50–99 and 100–199 cows were compared (OR = 5.5 *p* = 0.004 CI 1.7–17.6) and also when herds with 100–199 cows were compared to herds >400 cows (OR = 7.8 *p* = 0.014 CI 1.5–39.4). The mean within-herd prevalence also increased with herd size, being 13% in the smallest category (20–99 cows) and 56% in herds with >400 cows.

The study of Williams and Van Winde (13) showed that larger herd size is a risk factor for having a BoHV-1 positive herd status [OR = 1.005 *p* < 0.001 CI 1.003–1.007, per one cow increase in herd size (mean herd size 122.1)] in the United Kingdom.

Larger Irish herds (>99 cows) were more often seropositive compared to herds sized 31–65 cows (OR = 3.66 *p* < 0.001 CI 1.82–7.37) and to herds sized 66–99 cows (OR = 4.15 *p* < 0.001 CI 2.11–8.19), according to Sayers et al. (7). Also, in Ireland, Martinez-Ibeas et al. (4) found that larger herds (>99 cows) had a higher probability of having a recent circulation of BoHV-1 than smaller dairy herds (31–65 cows) (OR = 6.71 *p* = 0.015 CI 1.44–31.03). In the same study, also the chance of the herd status being positive for BoHV-1 was almost twice as high for larger herds (>99 cows) than smaller herds (31–65 cows) (OR = 1.8 *p* = 0.005 CI 1.19–2.75).

In a Dutch study by Van Wuijckhuise (12), in which 98% of all Dutch dairy herds were tested, it was found that the probability of herds having a negative or weakly positive bulk milk decreased linearly with herd size by a factor of 1.2 per 10 animals (OR = 0.84 *p* = <0.001 CI 0.84–0.85).

Bishop et al. (1) found that non-vaccinating Welsh dairy herds with positive bulk milk antibody titres to BoHV-1 (mean herd size 147) had significantly larger herd sizes (*p* < 0.01) than herds without antibodies (mean herd size 78).

Having a sizeable Spanish herd was a risk factor for being BoHV-1 positive (OR = 14.57 *p* = 0.004 CI 2.35–90.39) compared to smaller herds, in a study by Gonzalez-Garcia (3). Boelaert et al. (2) found a larger herd size in Belgium only to be a small risk factor (OR = 1.04 *P* = <0.001 CI 1.03–1.05).

O'Grady et al. (5) did not find a significant effect of herd size in Irish beef herds. Neither did Van Schaik et al. (9) and it was concluded that herd size was an indirect risk factor as the number of professional visits [e.g., by veterinarian, artificial insemination (AI) technician or cattle trader] is a measure of the herd size because these professionals visited large dairy herds more often than smaller dairy herds in the Netherlands.

#### Herd Type

The association between herd type, whether a herd contains solely dairy cattle, beef cattle or a mixture, and the presence of BoHV-1 in cattle herds was evaluated in three studies. In two of those, the type of the cattle holding was found to be significantly associated with BoHV-1 positivity.

Van Wuijckhuise et al. (12) found that Dutch herds that exclusively housed dairy cows, were almost twice more likely to have a negative or weakly positive bulk milk/BoHV-1 herd status than mixed herds (with beef or veal animals) (OR = 1.9 *p* = <0.001 CI 1.6–2.1). The same was found by Sayers et al. (7) when comparing BoHV-1 antibody-negative Irish dairy herds to those operating in mixed farming systems. The latter was over four times more likely to show signs of exposure to both BoHV-1 and bovine viral diarrhea virus (BVDV) (OR = 4.84 *p* = 0.024). The outcome for mixed herds with only a BoHV-1 infection was nearly significant (OR = 4.04 *p* = 0.071).

Boelaert et al. (2) found no differences in herd type, being dairy, beef or mixed in Belgium.

### Management Factors

#### Seasonal Calving

Only one study analyzed seasonal calving pattern and it was not found to be a risk factor. Sayers et al. (7) did not find differences in seroprevalence between spring-calving herds and all year-round calving herds in Ireland.

#### Presence of a Bull

The association between the presence of a bull in the herd and the presence of BoHV-1 was evaluated in three studies. In two studies this was found to be a risk factor.

In the United Kingdom, Williams and Van Winden (13) found the presence of a bull in the herd, or hired in on occasion, to be associated with an increased probability of positive BoHV-1 bulk milk (OR = 1.52 *p* < 0.005 CI 1.14–2.02). Also, Martinez-Ibeas et al. (4) found that Irish herds with more than one bull present were twice as likely to be categorized as BoHV-1 positive than those who had a single bull (OR = 2.13 *p* = 0.027 CI 1.08–4.19).

Van Schaik et al. (9) did not find differences between Dutch dairy herds that only used AI for service and those that used bulls for natural mating.

#### Borrowing Machinery

One study analyzed an operational activity on the farm and it was not found to be a risk factor. Van Schaik et al. (9) found that borrowing machinery from other farmers was not associated with BoHV-1 positive herds.

### Animal Factors

#### Breed

Two studies analyzed if the breed of cattle was a risk factor, only in one study this was found significant.

Gonzalez-Garcia et al. (3) found crossbreeding in Spanish beef herds between local breeds and Limousine or Charolais to be a significant risk factor (OR = 7.91 *p* = 0.001 CI 2.22–28.13). O'Grady et al. (5) did not find any differences between the breeding type of Irish beef herds.

#### Sex

Two studies analyzed if the sex of the animal was a risk factor, both confirmed this, with male cattle to be more of a risk.

Boelaert et al. (2) showed that bulls were more at risk to be seropositive than cows (OR = 1.37 *p* = 0.009 CI 1.08–1.74) in Belgium. A similar result was found by O'Grady et al. (5), a decreasing percentage of males within the beef herd was a significant protective factor among infected Irish herds (OR = 0.88 *p* = 0.04 CI 0.77–1.00), this converts to OR = 1.14 (1/0.88).

#### Age

The association between age of animals and the presence of BoHV-1 in cattle herds was evaluated in four studies. In three of those, older animals were found BoHV-1 positive significantly more often than younger animals, the fourth study did not find age to be a risk factor.

In Belgium, an increasing (centered) age was a risk factor for seropositivity, according to Boelaert et al. (2), but this effect leveled off at an older age (OR = 1.04 *p* = <0.001 CI 1.04–1.05). Martinez-Ibeas et al. (4) found that increasing age in Irish stock bulls was a risk factor for BoHV-1 seropositivity. Two-year-old bulls were five times more likely to be seropositive than 1-year-old bulls (OR = 5.15 *p* = 0.001 CI 1.89–14.03). For 3-year-old bulls (OR = 12.78 *p* = 0 CI 4.46–36.61) and 4-year-old bulls (OR = 28.94 *p* = 0 CI 9.35–89.5), this difference was even more distinct in comparison with 1-year-olds. Also, Raaperi et al. (6) found that the mean seroprevalence in cows was more than twice as high as that in youngstock in all Estonian herd size categories.

O'Grady et al. (5) did not find differences in age categories in a study on introducing beef bulls into a performance testing station in Ireland.

### Purchase Related Factors

#### Purchase of Cattle

The association between purchase and the presence of BoHV-1 in cattle herds was evaluated in ten studies. In seven of those, the introduction of new cattle was found to be a risk factor for BoHV-1 seropositivity.

Van Schaik et al. (9) found that purchase was a risk factor (OR = 1.32 *p* = 0.00 CI 1.15–1.52 per purchased cow). In this Dutch study, herds on average bought 6.6 cows a year. Purchase was also ranked as a risk factor (OR = 1.67 *p* = <0.001 CI 1.32–2.12) by Boelaert et al. (2) in Belgium for smaller herds (up to 50 animals per herd).

In the United Kingdom, Williams & Van Winden (13) found that the purchase of replacement cattle is a risk factor for the presence of BoHV-1 in bulk milk (OR = 2.83 *P* < 0.001 CI 2.15–3.74). They also found a significant difference in the mean amount of months since the last purchase, with BoHV-1 positive herds having purchased more recently (10.1 months) compared to BoHV-1 negative herds (19.6 months).

Martinez-Ibeas et al. (4) found that purchased bulls on dairy herds in Ireland were three times more likely to be seropositive for BoHV-1 than homebred bulls (OR = 3.08 *p* = 0.002 CI 1.51–6.29). Furthermore, this study revealed that bulls with a high number of movements between herds were more likely to be BoHV-1 seropositive (OR = 1.32 *p* = 0.019 CI 1.04–1.67). The average number of movements was 1.8 (range 1–7) and more movements meant higher chances of being seropositive. Herds with purchased bulls were approximately four times more likely to be categorized as having recent BoHV-1 circulation than herds where all the bulls were homebred (OR = 3.9 *p* = 0.039 CI 1.07–14.22). Herds with purchased bulls were almost three times more likely to have at least one positive bull in the herd than herds where all the bulls were homeborn (OR = 2.73 *p* = 0.009 CI 1.19–2.75).

Van Wuijckhuise et al. (12) found that the purchase of cattle was significantly associated with a negative or weakly positive BoHV-1 herd status, but there was an interaction between herd type and purchase of cattle. For Dutch herds with both dairy and beef/veal animals, there was a weak association between the purchase of cattle and a negative or weakly positive BoHV-1 status. For herds that exclusively housed dairy cows, the probability of having a negative or weakly positive BoHV-1 status decreased linearly by a factor of 1.3 per 10 animals purchased (OR = 0.79 *p* = <0.001).

Bishop et al. (1) found Welsh open dairy herds to have bulk milk antibodies to BoHV-1 a lot more often than closed herds (OR = 16.7 *p* < 0.05 CI 2.0–49.7). This was found for purchasing cattle in general, when looking specifically at hiring in bulls this was not significant, but there was a trend for herds practicing this natural mating strategy to be bulk milk positive (OR = 4.9 *p* = 0.08). Nor was it found significant whether purchased cattle were quarantined before introduction to the herd.

Gonzalez-Garcia et al. (3) concluded that external replacement was a massive risk factor in their predictive model in Spanish herds (OR = 116.78 *p* = 0.000 CI 14.94–912.33).

Three studies, Van Schaik et al. (10, 11) in the Netherlands and Raaperi et al. (6) in Estonia, did not find an association between purchase and BoHV-1 infection.

#### Rejected Export Cattle

Two studies analyzed if rejected export cattle or cattle not sold at a market that returned to their original herd was a risk factor, one confirmed this, the other not.

A Dutch study by Van Schaik et al. (11) analyzed rejected export cattle (or cattle not sold at a market) returning to the original herd and found this to be a significant risk factor (OR = 12.6 *p* = 0.03). However, in an earlier study, Van Schaik et al. (9) did not find this effect.

### Animal Contact Factors

#### Cattle Shows

The association between cattle shows and the presence of BoHV-1 in cattle herds was evaluated in four studies. In only one, participation was found to be significantly associated with BoHV-1 positivity.

Van Schaik et al. (9) found that participating in cattle shows was a risk factor for BoHV-1 infections (OR = 3.54 *p* = 0.05 CI 0.99–12.6). In later studies by Van Schaik et al. (10, 11), this effect was not found, neither was it found a significant risk factor in a study conducted by Gonzalez-Garcia et al. (3).

#### (Communal) Grazing

The association between (communal) grazing and the presence of BoHV-1 in cattle herds was evaluated in five studies. In two of those, pasture was found to be a risk factor for BoHV-1 positivity.

Van Schaik et al. (11) found that cattle grazing at other farms is a risk factor for the introduction of BoHV-1 among other diseases (OR = 7.0 *p* = 0.05). As opposed to indoor systems, open field keeping was considered a risk factor (OR = 3.07 *p* = 0.018 CI 1.29–7.29) by Gonzalez-Garcia et al. (3) in Spain, but if a communal aspect was practiced this was not a risk factor.

Raaperi et al. (6) did not find grazing to be a significant risk factor in Estonia. Twice, Van Schaik et al. (9, 10) did not find communal grazing a risk factor. These studies also analyzed the possibility of over-the-fence contacts with other cattle and neither found this to be a significant risk factor in the Netherlands.

#### Housing

One study analyzed housing on the farm and it was not found to be a risk factor. Raaperi et al. (6) studied several variables concerning housing, including keeping young stock together with cows, but did not find any significant factors in Estonia.

#### Other Species

One study analyzed other species and it was not found to be a risk factor. Gonzalez-Garcia et al. (3) studied the coexistence of sheep, goats, pigs and fattening calves on Spanish dairy and beef herds. The differences in BoHV-1 risk with and without other species were not significant.

### Neighborhood Factors

#### Herd Density

The association between herd density in a region and the presence of BoHV-1 in cattle herds was evaluated in six studies. Three of those found significant outcomes, both areas with a high and low density were found to be a risk factor.

O'Grady et al. (5) concluded that the increasing number of contiguous herds could reasonably be linked with biosecurity levels on the Irish beef study herds, given that infection risk is likely to increase with an increasing number of infected neighboring herds (OR = 1.13 *p* = 0.042 CI 1.01–1.33). Van Wuijckhuise et al. (12) found similar results, Dutch herds in areas containing <1 herd per square kilometer were 1.5 times more likely to have a negative or weakly positive bulk milk/BoHV-1 herd status than herds in areas with more than three herds per square kilometer (OR = 1.5 *p* = <0.001 CI 1.4–1.7). In this study, differences in numbers of animals per unit area were not significantly associated with BoHV-1 herd status.

Contrarily, herds in a lower dairy cattle dense region had a higher probability of being seropositive (OR = 2.8 *p* = 0.028 CI 1.11–7.01) according to Martinez-Ibeas et al. (4) in Ireland. For the seropositive bulls present in these regions, no significant differences were found (OR = 1.17 *p* = 0.49 CI 0.74–1.86). This finding about less densely populated Irish regions was met, only as a trend, by Sayers et al. (7). Herds in the least dairy dense part of Ireland (roughly the northern part of the country but not Northern Ireland) were found almost twice as likely to be categorized as positive as those in the densest region (roughly the southern part of the country) (OR = 1.77 *p* = 0.056 CI 0.98–3.18).

Boelaert et al. (2) found no differences in density of cattle or density of herds in Belgium related to BoHV-1. Neither did Gonzalez-Garcia et al. (3) find significant differences in herd density in Spain.

#### Distance Between Herds

Three studies analyzed if distance between herds was a risk factor, two of which found a significant association.

Each 100 meters distance between herds was found to decrease the risk to be BoHV-1 seropositive in the Netherlands (OR = 0.70 *p* = 0.00 CI 0.55–0.88) by Van Schaik et al. (9), this converts to OR = 1.43 (1/0.70). Proximity to an urban area was a risk factor in a Spanish study by Gonzalez-Garcia et al. (3) (OR = 7.58 *p* = 0.03 CI 1.21–47.24). Van Schaik et al. (10) reported the exact distance to the nearest other cattle herd and did not find differences between case (347 meters) and control herds (354 meters).

#### Escaping and Mingling

Three Dutch studies analyzed if escaping and mingling of cattle was a risk factor, one of which found a significant association.

The study by Van Schaik et al. (10) found the escape and mingling of milking cows with other cattle to be a risk factor (OR = 6.85 *p* = 0.05). Moreover, in this same study, the risk factor “young stock escapes” was separately assessed and not significant. In two other studies of Van Schaik et al. (9, 11) escaping and mingling of cattle was not found significant.

### Indirect Risk Factors

#### Visitors

The association of different aspects of visitors and the presence of BoHV-1 in cattle herds was evaluated in five studies. In three of those, visitors were found to be a risk factor.

Indirect iatrogenic spread was proposed by Raaperi et al. (6) in Estonia. The probability for high within-herd prevalence was higher in farms where the veterinarian was an employee (OR = 6.05 *p* = 0.03 CI 1.19–30.62) and where the AI technician was an employee (OR = 5.54 *p* = 0.04 CI 1.10–27.91). The study of Van Schaik et al. (10) showed that the use of protective clothing by professional visitors (e.g., veterinarians, AI technicians) tended to be a preventive factor against the introduction of BoHV-1 (OR = 0.43 *p* = 0.06). 73% of case herds (outbreak herds) did not have or did not always use protective clothing. A Dutch cohort study by Van Schaik et al. (11) following 95 SPF (Specific Pathogen Free) dairy herds over 2 years substantiated the previous finding that wearing protective clothing by professional visitors was a protective factor (OR = 0.2 p = 0.004), this converts to OR = 5.0 (1/0.2). In this study, three of the four outbreak herds did not provide protective clothing to visitors. However, in an earlier study by Van Schaik et al. (9), neither the use of protective clothing, temporary workers nor the number of visits per year by AI technicians were found to be significant.

Not only professional visitors are a risk for introduction. Also, occasional visitors (at least once a week), such as neighbors, family and friends in the barn, are a risk factor (OR = 4.06 *p* = 0.02 CI 1.28–12.9) as described by Van Schaik et al. (9).

#### Vaccination

Two studies analyzed vaccination and neither found an association. Sayers et al. (7) did find a trend for BoHV-1 positive Irish herds to vaccinate more often than negative herds (OR = 31.88 *p* = 0.057 CI 0.92–1,102.57). It was however concluded that herds vaccinating for BoHV-1 were significantly more likely to also vaccinate for BVDV (OR = 3.63 *p* = 0.012) and that larger herds were more likely to vaccinate for BoHV-1. Herds with >99 cows were vaccinated far more often than smaller herds with an average 31–65 cows (OR = 15.11 *p* = 0.009). Also, there was a trend in vaccination patterns between herds with an average size of 66–99 cows and herds with a smaller size of on average 31–65 cows (OR = 7.74 p=0.055). Another trend was that non-spring calving herds vaccinated for BoHV-1 more often (OR = 2.40 *p* = 0.067).

Raaperi et al. (6) did not find any relation between herd prevalence of BoHV-1 and vaccination history or vaccination for diseases other than IBR or BVD.

## Discussion

This literature review confirmed that many risk factors can play a role in introducing BoHV-1 into a cattle herd. All studies used presence of antibodies as measure for infection, which is correlated with introduction of the virus. Risk factors in one country may not have the same importance in another country. The choice to limit the review to European countries was made in order to facilitate comparison.

For this literature review, studies were included that quantified the risk factors by OR to facilitate comparison of the results. When searching for other measures to quantify risk factors, just one additional study was found which used hazard ratios (HR). However, the survival analysis in this study of Van Schaik et al. (8) was based on the same data as used for the logistic regression of Van Schaik et al. (9) in which OR of the risk factors were reported. For the sake of comparability and because the results were fairly similar, we decided to only report the OR.

Some risk factors were only studied in a limited number or even a single study. These results should be especially interpreted with prudence.

Most studies were based on questionnaires to obtain information on possible risk factors. In these studies, measures were taken to get representative answers, such as minimizing recall bias and conducting interviews by as few persons as possible. Risk factors were not always significantly associated with the outcome variable seropositivity for BoHV-1. Farmers may not have responded properly about practices and provided socially desirable answers especially about some commonly known risk factors.

A total of four studies that were included in this review were performed in the Netherlands. This relatively high number of studies compared to other European countries was because a compulsory BoHV-1 eradication campaign was in place in the Netherlands for a short period from 1998 to 1999. It was canceled due to vaccine contamination issues. Much scientific research was done in the customization and aftermath of the CP. Since then, the average herd size almost doubled, there is more import of live cattle from other countries, herds purchase cattle more often, and there is a growing number of herds that have their young stock raised in specialized young stock raising herds. These changes may hamper extrapolation of the study findings from the nineties to current times. Also, multiple Irish studies were included in the review. These BoHV-1 studies were performed in the development of a national CP for BVDV. Along with data collection for BVDV, the studies often simultaneously investigated BoHV-1. Therefore, this literature review mostly covers the cattle situation in Northwestern Europe. The discrepancy in results between studies in general, but certainly in those performed in the same country can be explained by the fact that risk factors can disappear when (biosecurity) measures are implemented or when prevalence reduces to low levels, generating a lack of statistical power. In general, changing national cattle legislation or other (inter)national circumstances can influence risk factors. For example, the purchase of cattle may be driven by economic incentives or other external drivers that affect herd composition, and therefore the importance of this risk factor may increase or decrease.

Most papers found herd size to be positively associated with BoHV-1 herd infection. Several studies excluded very small holdings. Larger herds have more contacts that can introduce the virus into the herd (e.g., more professional visitors and more purchased cattle for replacement). Additionally, the purchase of cattle into a herd is often required to achieve this larger herd size. An infection with BoHV-1 is also easier maintained in a large herd. In smaller herds, the number of susceptible animals is lower, so infections may not be preserved. The range in average herd size in the northwestern part of Europe is quite similar between countries. Extremely large herds with thousands or even ten thousand cattle such as, for example, in North and South America or the Middle East do not exist. Often, underlying management or herd structure related to herd size will be the real risk factor for the BoHV-1 status rather than herd size alone. Herd size is therefore considered a proxy for other interlinked risk factors.

Studies indicating herd type (dairy or beef) were not conclusive, as both types were found to have an increased risk of being infected with BoHV-1. Overall, there was a slight tendency for beef herds to be BoHV-1 positive more often. These type of animals are often more traded, which could explain the higher risk as well as other risk factors that may be linked to herd type and are discussed below.

Whether the sex of cattle is a risk factor or not is not widely documented. Bulls have been found to have a higher risk of becoming BoHV-1 positive. Bulls have more changing contacts compared to cows. Additionally, beef bulls more frequently participate in cattle shows and bulls are more often purchased from other herds. Also, bulls possibly display more risky behavior than cows. Escaping and mingling was found to be a risk for virus introduction into herds. Since BoHV-1 is also a venereal transmissible disease (IPV/IPB), it could be expected that bulls play a role with natural service by these means, but differences between natural breeding and AI were not reported by any of the studies. In the past, BoHV-1 positive semen used for AI was a well-known source of the introduction of BoHV-1. Due to strict measures for AI companies, nowadays, semen is guaranteed BoHV-1 free, which explains the fact that an association between AI and BoHV-1 was no longer found.

Age of cattle was found to be a risk factor for BoHV-1, but can be considered a proxy for potential exposure time. Antibodies are kept lifelong, with BoHV-1 also generating lifelong latency of the virus and thereby risk of reactivation. This was confirmed by the fact that studies showed that in positive herds, older cattle most commonly have antibodies against BoHV-1. Contacts between adult cattle are therefore riskier than contacts with young stock. Since seroprevalence in dairy herds is often found to be age-dependent, this is a plausible explanation. Surprisingly, keeping young stock and cows together was not a risk factor, which may be explained by lower stress levels given the unchanging environment. This also underlines that although BoHV-1 inflicts respiratory illness, the virus is not easily transmitted aerogenically over larger distances. Likely, the spread of the virus from cows to young stock is more dependent on indirect viral transmission routes related to herd management. Feeding residual cattle-fodder to other age-cohorts of cattle on the farm may be an example of this. Also, housing different age-cohorts in multiple buildings may counteract virus spread. Ongoing cattle replacement from own stock as a standard management procedure ensures outgrowth of the positive age-cohorts in the absence of reactivation or reintroduction. Age was also found to have an effect when looking at the break out of cattle, in the same study it was found a significant risk factor for adult milking cows, but not for young stock.

Purchase was considered to be any cattle brought into the herd from another farm, although the definition was not clearly stated in every study. The findings on purchased cattle highlight that farmers should consider the antibody BoHV-1 status of cattle before transportation to prevent concomitant introduction. After arrival, a quarantine period may limit spread of infection (when introduced), but is not common practice in daily cattle routines. In general, a closed farming system and the use of protective clothing for (professional) visitors can, to a large extend, minimize the risk of BoHV-1 introduction. Progress on this matter can be made for all professional visitors that come in direct contact with cattle. A measure that may facilitate awareness is to publish the BoHV-1 herd status of farms to adjacent farmers and professional visitors so that extra biosecurity measures can be taken to prevent infection with BoHV-1 from an infected neighboring herd. Known herd status also promotes purchasing cattle from certified BoHV-1 free herds because provenance can be checked in advance. Otherwise, if unknown, testing cattle before movement can largely reduce the risk of introduction. Most studies indicated the introduction of latently infected cattle as a common way of BoHV-1 transmission between herds. To a lesser extent purchase of acutely infected cattle also plays a role. Movement and mixing of cattle will be stressful, resulting in higher chances of reactivation of the virus in latently infected cattle. Studies that did not find a relationship with purchase often had a limited number of cattle purchased during the study. The risk of purchase is sometimes underestimated by farmers, as they consider themselves a closed herd that never buy female cattle. The rare or sporadic purchase of a breeding bull is not perceived as impacting their closed herd status and is somewhat overlooked in maintaining a closed herd.

One study found an extremely high multivariable estimated OR (116.78) for the risk factor purchase of replacement cattle. This seems unreliable, especially since it univariably had an associated value of OR = 2.74. Although this unusually high OR is not discussed at all in the original paper itself, it should be interpreted with care and therefore it is not presented in the summarized results in Table 1.

Cows returning from markets or rejected for export were found a risk factor in the Dutch situation in one study about 20 years ago. The cattle industry's infrastructure has changed since then because of altered legislation due to the foot-and-mouth disease (FMD) outbreak the country faced in 2001. The risk of transportation will still exist, but cattle returning to their herds after being initially sold, is rare to non-existent nowadays. In fact, markets no longer exist. In addition, export, import and show cattle are often quarantined and tested for their BoHV-1 status, which leads to a minimized risk for those risk factors.

Communal grazing was found to be a risk factor in two out of five studies that investigated the risk of grazing for having BoHV-1 positive animals in a herd or for a herd to be BoHV-1 seropositive. This type of pasturing is not common anymore in all countries, but it, for example, still occurs in some mountainous areas in the summertime. The extensive way of keeping cattle this way will generate less stress and possibly limit the risk of reactivation and transmission of the virus. Also, calving being a known trigger for reactivation, will in most of these systems have occurred before moving to the pasture, thereby creating less of an effect since the cow will no longer be infectious. As a comparison, for several years, young stock raising as a separate farm business has proliferated in Europe as it is long term common in North America. Calves are sent to these specialized herds, and the animals return to the original herd as raised pregnant heifers. The risk this management brings along will be more considerable when the young stock raiser operates for multiple herds and does not assess BoHV-1 status.

It was concluded in one study that sheep and other animals are a negligible risk factor for having BoHV-1 in a herd. More research focusing on these contacts would be worthwhile, since farmers tend to externalize reasons for introducing the virus into their herds. Often, factors they cannot influence, are considered important, such as small and wild ruminants, but also interference with other species (e.g., birds). When housed on the same farm, the amount of contact of cattle with small ruminants varies a lot between countries. However, countries that imposed CPs and became free of BoHV-1 (e.g., Germany and the Scandinavian countries) did so without including regulations on small and wild ruminants.

Herd density and distance to neighboring herds were found to be a risk factor in several studies. The risk may be explained by underlying factors such as air currents, visits of neighboring farmers or children, professional workers and visitors, contacts between cattle of neighboring herds, contacts with other animals (cats, dogs, mice, rats, etc.), borrowing machinery and vehicular movements between proximal farms. One study found that closeness to an urban area increases the chances of seropositivity. However, closeness to an urban area was positively correlated with distance to other cattle herds. The study was carried out in an otherwise low-density herd area, where only herds in urban areas were relatively close to each other. In Ireland, the contrary was reported in two studies in that herds in the least dairy dense part of the country were more often positive for BoHV-1. This was proposed due to a higher proportion of beef cattle in these regions and less implementation of biosecurity measures in these herd types, so in fact the area was still cattle dense.

Veterinarians or AI technicians employed on the farm were found to be a risk factor for BoHV-1. This seems unexpected because veterinarians or AI technicians that visit multiple, different farms daily in their ambulatory work would likely carry more risk. An explanation could be that when veterinarians or AI technicians are employees of the farm, they might work at multiple, intensive sites and there probably is a tendency to handle cattle more frequently for diagnostic purposes, perform invasive treatments and heat detection compared to those where these professionals visit a herd on call. Still, it may be expected that fulltime employees are more focused on biosecurity. Iatrogenic spread of the virus will facilitate within herd transmission. Employment by farms of a veterinarian or AI technician is likely related to herd size, so may also be a confounder for increased transmission within larger herds. Several Dutch studies have investigated biosecurity in relation to introductions of infectious diseases. The herds free from disease had less risky contacts than herds with outbreaks. Moreover, the review showed that biosecurity around visitors is essential, professional visitors should be convinced to wear protective clothing supplied by the farmer before handling cattle at all times.

BoHV-1 seroprevalence data should always be interpreted with caution since conventional IBR vaccines (non-marker) were and are widely used in many European countries. Most studies took vaccination data into account. Depending on the country where and when the study was performed, it should be considered that cattle might be vaccinated with conventional vaccine earlier in life, thus interfering with diagnostics (no distinction in detected antibodies derived from natural infection or vaccination possible). Vaccination is often initiated after the introduction of infection and not always as a preventive management tool. Therefore, vaccination can be considered as an aggregate indicator for underlying risk factors for introduction of BoHV-1.

Four studies (1, 6, 7, 13) found associations between BoHV-1 and presence of other infectious diseases. All four reported that herds positive in bulk milk for BVDV antibodies were significantly more likely to also be positive for BoHV-1 (range in OR 2.31–12.0). One study mentioned the same for Leptospirosis (OR = 7.5). Although these diseases are thereby presented as a risk factor for BoHV-1 positivity, it probably just indicates that there are common risk factors in these herds related to introduction of infectious diseases into the herds.

## Conclusions

This study describes the most relevant risk factors for the introduction of BoHV-1 in cattle herds based on literature findings of consistently high odds ratios. Risk factors most often found to facilitate a BoHV-1 infected herd were herd size, purchase of cattle, cattle density, age of cattle, distance to neighboring cattle herds and professional visitors. When eradication is considered on a national, regional or even herd level, mitigating the risk of these factors should be taken into account. Other animal species (e.g., sheep) are likely of negligible risk. The findings should also be used when educating and communicating with farmers, veterinarians and other professional visitors about reducing the risk of contracting an infection with BoHV-1.

Biosecurity measures that mitigate this risk are keeping a closed herd; when purchase is necessary then acquire cattle from known BoHV-1 free herds or screen in advance for presence of BoHV-1 antibodies; rearing own young stock; provide protective farm clothing (coverall and boots); prohibit direct and lengthy animal contact with other cattle from herds through grazing or escaping and mingling; implementation of testing schemes for cattle participating in shows.

It is normal practice to concentrate on the most impactful factors in the early stages of disease control programmes (CP) to make tangible progress and gain stakeholder momentum. Therefore, for the implementation of CPs, it is crucial to know which risk factors related to virus introduction or reactivation need to be prioritized. In the early stages of designing a CP, modeling can assess the epidemiological potential of different control scenarios for BoHV-1, and the results of this review could be used as input for such models.

## Data Availability Statement

Publicly available datasets were analyzed in this study. This data can be found at: PubMed & Web of Science.

## Author Contributions

All authors listed have made a substantial, direct and intellectual contribution to the work, and approved it for publication.

## Funding

This literature review was partly funded by PPS 1H4F (Public and Private Cooperation Project 1Health4Food) financed by the Ministry of Agriculture, Nature and Food Safety (LNV, The Hague, The Netherlands) and the producers organizations for dairy (ZuivelNL, The Hague, The Netherlands) and veal (SBK, Zeist, The Netherlands).

## Conflict of Interest

The authors declare that the research was conducted in the absence of any commercial or financial relationships that could be construed as a potential conflict of interest.

## Publisher's Note

All claims expressed in this article are solely those of the authors and do not necessarily represent those of their affiliated organizations, or those of the publisher, the editors and the reviewers. Any product that may be evaluated in this article, or claim that may be made by its manufacturer, is not guaranteed or endorsed by the publisher.
